# Gestational Tissue-Derived Human Mesenchymal Stem Cells Use Distinct Combinations of Bioactive Molecules to Suppress the Proliferation of Human Hepatoblastoma and Colorectal Cancer Cells

**DOI:** 10.1155/2019/9748795

**Published:** 2019-07-04

**Authors:** Nitchapon Paiboon, Witchayaporn Kamprom, Sirikul Manochantr, Chairat Tantrawatpan, Duangrat Tantikanlayaporn, Sittiruk Roytrakul, Pakpoom Kheolamai

**Affiliations:** ^1^Center of Excellence in Stem Cell Research, Faculty of Medicine, Thammasat University, Pathumthani 12120, Thailand; ^2^Department of Clinical Microbiology and Applied Technology, Faculty of Medical Technology, Mahidol University, Bangkok 73170, Thailand; ^3^Division of Cell Biology, Faculty of Medicine, Thammasat University, Pathumthani 12120, Thailand; ^4^Proteomics Research Laboratory, Genome Institute, National Science and Technology Development Agency, Pathumthani 12120, Thailand

## Abstract

**Background:**

Cancer has been considered a serious global health problem and a leading cause of morbidity and mortality worldwide. Despite recent advances in cancer therapy, treatments of advance stage cancers are mostly ineffective resulting in poor survival of patients. Recent evidences suggest that multipotent human mesenchymal stem cells (hMSCs) play important roles in growth and metastasis of several cancers by enhancing their engraftment and inducing tumor neovascularization. However, the effect of hMSCs on cancer cells is still controversial because there are also evidences demonstrating that hMSCs inhibited growth and metastasis of some cancers.

**Methods:**

In this study, we investigated the effects of bioactive molecules released from bone marrow and gestational tissue-derived hMSCs on the proliferation of various human cancer cells, including C3A, HT29, A549, Saos-2, and U251. We also characterized the hMSC-derived factors that inhibit cancer cell proliferation by protein fractionation and mass spectrometry analysis.

**Results:**

We herein make a direct comparison and show that the effects of hMSCs on cancer cell proliferation and migration depend on both hMSC sources and cancer cell types and cancer-derived bioactive molecules did not affect the cancer suppressive capacity of hMSCs. Moreover, hMSCs use distinct combination of bioactive molecules to suppress the proliferation of human hepatoblastoma and colorectal cancer cells. Using protein fractionation and mass spectrometry analysis, we have identified several novel hMSC-derived factors that might be able to suppress cancer cell proliferation.

**Conclusion:**

We believe that the procedure developed in this study could be used to discover other therapeutically useful molecules released by various hMSC sources for a future *in vivo* study.

## 1. Background

Cancer has been considered a serious global health problem and a leading cause of morbidity and mortality worldwide. While an early diagnosed cancer can be cured by surgery or radiotherapy, patients with an advance stage of cancer can only be treated with chemotherapeutic agents or immunotherapy. Despite significant improvements during the past decades, the effectiveness of those treatments, especially in patients with solid tumors, is limited resulting in the poor survival rate of those patients.

Several recent evidences suggest that human mesenchymal stem cells (hMSCs) play important roles in growth and metastasis of various cancer cells and affect their responses to chemotherapeutic agents. hMSCs are multipotent stem/progenitor cells that exist in various tissues, such as bone marrow, adipose tissue, umbilical cord, placenta, and chorion [1–5]. Due to their ability to produce and release bioactive molecules that have various therapeutic potentials, hMSCs have been considered potential cell sources for many clinical applications [6, 7].

Previous studies show that MSCs enhanced the engraftment rate of breast cancer, ovarian cancer, melanoma, glioma, and colon cancer cells in animal models. Some studies also demonstrate that MSCs could migrate from circulation into cancer tissues and become cancer-associated fibroblasts (CAFs) and pericytes. Those MSC-derived CAFs and pericytes then released several proangiogenic factors that induce tumor neovascularization leading to the rapid tumor growth and metastasis [8–16]. The immunomodulatory property of MSCs is also believed to promote tumor growth by reducing immune reaction against tumor cells [17].

Despite those evidences, there are other studies demonstrating that MSCs inhibited growth and metastasis of several cancers, including colon cancer, hepatoma, and melanoma [18–20]. Those conflicting results possibly arise from the variability of both MSC sources and cancer cell types used in those studies.

Although bone marrow-derived hMSCs (BM-hMSCs) have been the standard source of hMSCs for most research and clinical applications, their harvest requires an invasive procedure and their number declines with age [21, 22]. Therefore, gestational tissue-derived hMSCs which can easily be obtained in large quantity by a non-invasive procedure have been considered more suitable sources of hMSCs for clinical applications. However, types of bioactive molecules that are released from gestational tissue-derived hMSCs and their effects on the properties of cancer cells have yet to be characterized. Therefore, the present study is aimed at comparing the effects of several gestational tissue-derived hMSCs, including placenta-derived hMSCs (hAMSCs), chorion-derived hMSCs (CH-hMSCs), and umbilical cord-derived hMSCs (UC-hMSCs), on the proliferative capacity of five distinct human cancer cells (hepatoblastoma cell C3A, colon adenocarcinoma cell HT29, lung adenocarcinoma cell A549, osteosarcoma cell Saos2, and glioma cell U251) with that of bone marrow-derived hMSCs (BM-hMSCs) using an *in vitro* model. We also identified the hMSC-derived factors that inhibit cancer cell proliferation by using protein fractionation and mass spectrometry analysis.

## 2. Methods

### 2.1. Subjects

This study was approved by the ethical committee for human researches, Faculty of Medicine, Thammasat University, which was in accordance with the Declaration of Helsinki, the Belmont Report, and ICH-GCP. Human bone marrow samples were obtained from three healthy volunteers. The human gestational tissues of normal pregnancies (umbilical cord, placenta, and chorion) were obtained from three healthy women after labor. All donors gave written informed consent.

### 2.2. Isolation and Culture of hMSCs

hMSCs were isolated and cultured as described in our previous study [23]. Bone marrow-derived hMSCs were isolated using Ficoll-Hypaque (Robbins Scientific Corporation, USA) density gradient centrifugation and cultured in Dulbecco's modified Eagle medium (DMEM) (GIBCO™, Invitrogen Corporation, USA) supplemented with 10% (*v*/*v*) fetal bovine serum (FBS) (Lonza, USA), 100 U/ml penicillin, and 100 *μ*g/ml streptomycin. The cell suspensions at a density of 2 × 10^5^ cells/cm^2^ were cultured in a 25 cm^2^ culture flask (Corning, USA). For gestational tissue-derived hMSC isolation, umbilical cord, fetal side of the placenta, and chorion (chorionic membrane) were manually separated and cut into small pieces and incubated with 0.25% (*w*/*v*) trypsin-EDTA (GIBCO™, Invitrogen Corporation, USA) for 30 minutes at 37°C. The pieces were washed twice with PBS, resuspended in DMEM+10% (*v*/*v*) FBS and cultured in a 25 cm^2^ culture flask (Corning, USA). Cells were cultured at 37°C, and the medium was changed every 3 days. Cells were passaged when they reached 80% confluence. The morphological features of hMSCs were observed and photographed under an inverted microscope (Nikon Eclipse Ts2R, Japan).

### 2.3. Culture of Human Cancer Cells

Hepatoblastoma cell C3A, osteosarcoma cell Saos2, lung adenocarcinoma cell A549, and glioma cell U251 were cultured in DMEM (GIBCO™, Invitrogen Corporation, USA) supplemented with 10% (*v*/*v*) fetal bovine serum (FBS) (Lonza, USA) while colon adenocarcinoma cell HT29 was cultured in DMEM/F12 supplemented with 10% (*v*/*v*) FBS (Lonza, USA). Cells were cultured at 37°C, and the medium was changed every 3 days. Cells were passaged when they reached 80% confluence.

### 2.4. Characterization of Cultured hMSCs by Flow Cytometry

The cells were harvested and immediately processed for flow cytometric analysis as described in our previous study [23]. Briefly, hMSCs (passages 3^rd^-5^th^) were washed twice with PBS. 4 × 10^5^ hMSCs were then resuspended in 50 *μ*l PBS, incubated with 10 *μ*l fluorochrome-labeled mouse anti-human monoclonal antibodies: anti-CD45-FITC (BD Pharmingen, USA), anti-CD34-PE (Biolegend, USA), anti-CD90-FITC (AbD Serotec, USA), anti-CD73-PE (BD Pharmingen, USA), and anti-CD105-PE (Miltenyi Biotec, Germany) for 30 minutes at 4°C in the dark. After being incubated with the antibodies, cell pellets were washed twice with PBS and fixed with 1% (*w*/*v*) paraformaldehyde in PBS. Flow cytometry was performed by FACSCalibur™ Flow cytometer using CellQuest™ software (Becton Dickinson, USA).

### 2.5. Osteogenic and Adipogenic Differentiation of hMSCs

hMSCs (passages 3^rd^-5^th^) were used to assess their adipogenic and osteogenic differentiation potentials as described in our previous study [23]. For adipogenic differentiation, 5 × 10^4^ hMSCs were cultured in NH AdipoDiff® Medium (Miltenyi Biotec, Germany). The medium was changed every 3 days. After 3 weeks of growth in adipogenic medium, the cells were washed twice with PBS, fixed with 10% formaldehyde for 15 minutes, and rinsed with distilled water. Then, the cells were incubated with Oil Red O solution [0.5% (*w*/*v*) in isopropanol] for 20 minutes at room temperature and observed under an inverted microscope (Nikon Eclipse Ts2R, Japan).

For osteogenic differentiation, 5 × 10^4^ hMSCs were cultured in NH OsteoDiff® Medium (Miltenyi Biotec, Germany). The medium was changed every 3 days. After 3 weeks of growth in osteogenic medium, the cells were fixed with 4% paraformaldehyde, incubated with 40 mM Alizarin Red S solution (Sigma Aldrich, USA) for 20 minutes at room temperature, and observed under an inverted microscope (Nikon Eclipse Ts2R, Japan).

### 2.6. Preparation and Fractionation of hMSC-Conditioned Media

hMSC-conditioned media were prepared as described in our previous study [23]. 7 × 10^5^ hMSCs (passages 3^rd^-5^th^) were cultured in DMEM+10% FBS for 24 hours. Then, the cells were washed twice with 10 ml sterile PBS and incubated with 15 ml serum-free medium (SFM) which is MesenCult™-ACF Plus Medium (STEMCELL technologies, USA) for further 24 hours. To prepare cancer-associated hMSC-conditioned media, 5 × 10^4^ hMSCs were co-cultured with 5 × 10^4^ human cancer cells using a transwell culture system for 7 days. After co-culture, the transwell inserts containing human cancer cells were removed; the hMSCs were washed twice with 5 ml sterile PBS and incubated with 600 *μ*l SFM for further 24 hours.

After incubation, the conditioned media were collected, centrifuged at 2000 rpm for 10 minutes at 4°C, and filtered through a 0.45 *μ*m syringe filter (Corning, USA). The filtered conditioned medium was then fractionated into 5 distinct fractions according to the molecular weight of their protein composition using ultraspin columns with molecular weight cutoff (MWCO) at 100 kDa (Pall Corporation, USA), 50 kDa (Merck, Germany), 30 kDa (Pall Corporation, USA), and 10 kDa (Pall Corporation, USA) as described in our previous study [23]. To fractionate hMSC-conditioned medium, the medium was transferred to 100 kDa ultraspin columns and centrifuged at 4500 rpm for 15 minutes at 4°C. After centrifugation, the fraction of hMSC-conditioned medium retained in the column was collected while the flow through was transferred to the 50 kDa ultraspin columns for further centrifugation. By repeating this procedure with the 30 kDa and 10 kDa columns, the hMSC-conditioned medium was successfully fractionated into 100 kDa, 50 kDa, 30 kDa, 10 kDa, and less than 10 kDa (<10 kDa) fractions.

### 2.7. Effects of hMSC-Derived Bioactive Molecules on the Proliferation and Migration of Cancer Cells

For proliferation assay, the number of cancer cells was monitored by the real-time cell analysis (RTCA) of a cell culture system as described in our previous study [23]. Briefly, 2.5 × 10^4^ cancer cells were added into an individual well of culture E-Plate (Roche Applied Science, USA) containing 100 *μ*l normal or cancer-associated hMSC-conditioned media. The E-Plates were incubated at 37°C and monitored on the RTCA system by the xCELLigence real-time cell analyzer (Roche Applied Science, USA) at 5-minute time intervals for 7 days. The number of cancer cells in each well was continuously monitored throughout the entire culture period and reported as the cell index. Cancer cells cultured in DMEM+10% FBS served as controls.

For migration assay, hMSC-conditioned media were used to induce migration of hepatoblastoma C3A cells through 8 *μ*m transwell (Costar, Corning, USA) as described in our previous study [24]. 1 × 10^6^ C3A cells were seeded in the transwell inserts which were placed into wells of the 24-well plate (Costar, Corning, USA) containing 600 *μ*l of various hMSC-conditioned media. After 6 hours of culture, the numbers of C3A cells that migrate to the other side of the transwell's membrane were determined by hematoxylin staining. C3A cells cultured in C3A-conditioned medium served as controls.

### 2.8. Migratory Ability of hMSCs toward Cancer Cells

Cell migration ability was assessed using a 24-well transwell chamber (Corning, USA) with a 8 *μ*m pore polyester membrane insert as described in our previous study [23, 24]. Briefly, 5 × 10^4^ C3A or HT29 cells were plated into the lower chamber of transwell containing 600 *μ*l DMEM+10% FBS and incubated at 37°C for 24 hours to allow cell attachment. After that, the medium was changed to 600 *μ*l DMEM supplemented with 2% (*v*/*v*) FBS and the cells were cultured for further 24 hours. The following day, 4 × 10^4^ hMSCs were seeded into the upper chamber of the transwell inserts and cultured for further 6 hours. At the end of culture, cells that migrate to the other side of the transwell membrane were stained with hematoxylin and counted from at least ten randomly selected microscopic fields. The hMSCs cultured in transwell whose lower chamber contained cell-free DMEM+2% FBS served as controls.

### 2.9. Mass Spectrometry Analysis and Protein Identification

Mass spectrometry analysis was performed as described in our previous study [24]. The proteins were digested by trypsin and analyzed by ESI ion trap mass spectrometry. Identification and quantification of each protein were performed by DeCyderMS differential analysis software 2.0 (GE Healthcare, USA) and MASCOT search engine software (Matrix Science, UK) based on NCBInr human protein databases. The identified proteins were then categorized into non-secretory, classical secretory, and non-classical secretory proteins by SignalP and SecretomeP software (Center for Biological Sequence Analysis; CBS, DK). Finally, the secretory proteins were further categorized by PANTHER and UniPortKB software into separated groups according to their functions.

### 2.10. Statistical Analysis

Data were presented as the mean ± standard error of the mean (SEM). The Mann-Whitney *U* test was used to assess the significance of differences between observed data. *P* < 0.05 was considered to be statistically significant.

## 3. Results

### 3.1. Characteristics of hMSCs Derived from Bone Marrow and Gestational Tissues

**Figure 1:**
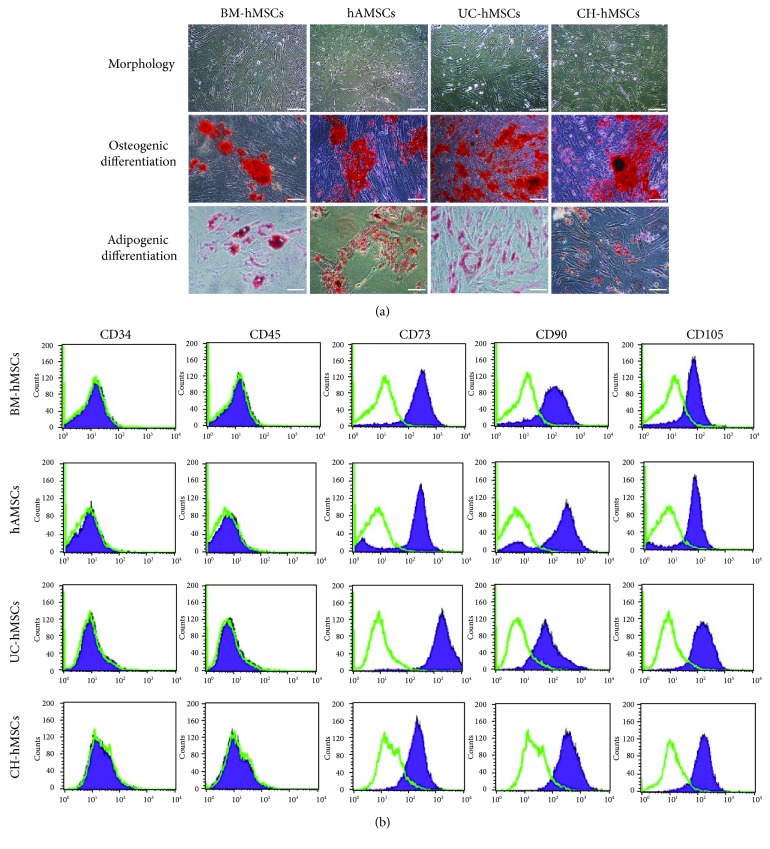
Characteristics of bone marrow- and gestational tissue-derived hMSCs. (a) Morphology, adipogenic, and osteogenic differentiation of bone marrow- and gestational tissue-derived hMSCs (scale bar = 200 *μ*m). (b) Immunophenotypes of bone marrow- and gestational tissue-derived hMSCs as determined by flow cytometry.

hMSCs derived from gestational tissues, including the fetal side of the placenta (hAMSCs), umbilical cord (UC-hMSCs), and chorion (CH-hMSCs), exhibited similar characteristics to those of bone marrow-derived hMSCs (BM-hMSCs). The gestational tissue-derived hMSCs displayed fibroblast-like morphology and could differentiate to adipocytes and osteocytes as demonstrated by Oil-Red O and Alizarin Red S staining, respectively (Figure 1(a)). Moreover, the gestational tissue-derived hMSCs also expressed typical hMSC surface markers (positive for CD73, CD90, and CD105 and negative for hematopoietic markers CD34 and CD45; Figure 1(b)).

### 3.2. Effect of hMSC-Derived Bioactive Molecules on Cancer Cell Proliferation

**Figure 2:**
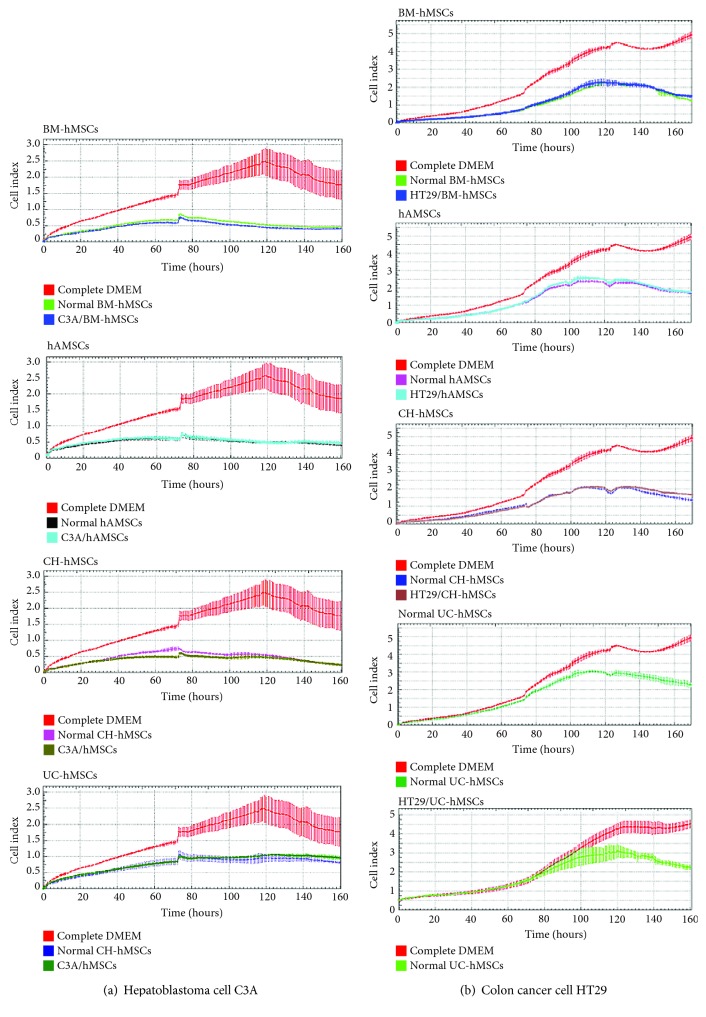
Effects of hMSC-derived bioactive molecules on the proliferation of C3A and HT29 cell. Graphs show the growth kinetics of hepatoblastoma cell C3A (a) and colon adenocarcinoma cell HT29 (b), which were cultured in conditioned medium derived from various normal and cancer-associated hMSC-conditioned media for 7 days. The growth kinetics of cancer cells were determined by the xCELLigence real-time cell analyzer and were reported as the cell index against culture time. C3A cells cultured in DMEM supplemented with 10% (*v*/*v*) FBS and HT29 cells cultured in DMEM/F12 supplemented with 10% (*v*/*v*) FBS serve as controls. Data are presented as the mean ± SEM of three independent experiments.

To study the effect of hMSC-derived bioactive molecules on cancer cell proliferation, the conditioned media derived from various hMSC sources were used to culture 5 human cancer cell lines, C3A, HT29, Saos2, A549, and U251. The condition media derived from all normal hMSC sources significantly reduced the proliferation of both hepatoblastoma cell C3A and colon adenocarcinoma cell HT29 (Figures 2(a) and 2(b)). The inhibitory effect of bone marrow- and gestational tissue-derived hMSCs on C3A and HT29 cell proliferation was observed at approximately 10 hours after culture and was maintained for the entire culture period (Figures 2(a) and 2(b)).

**Figure 3:**
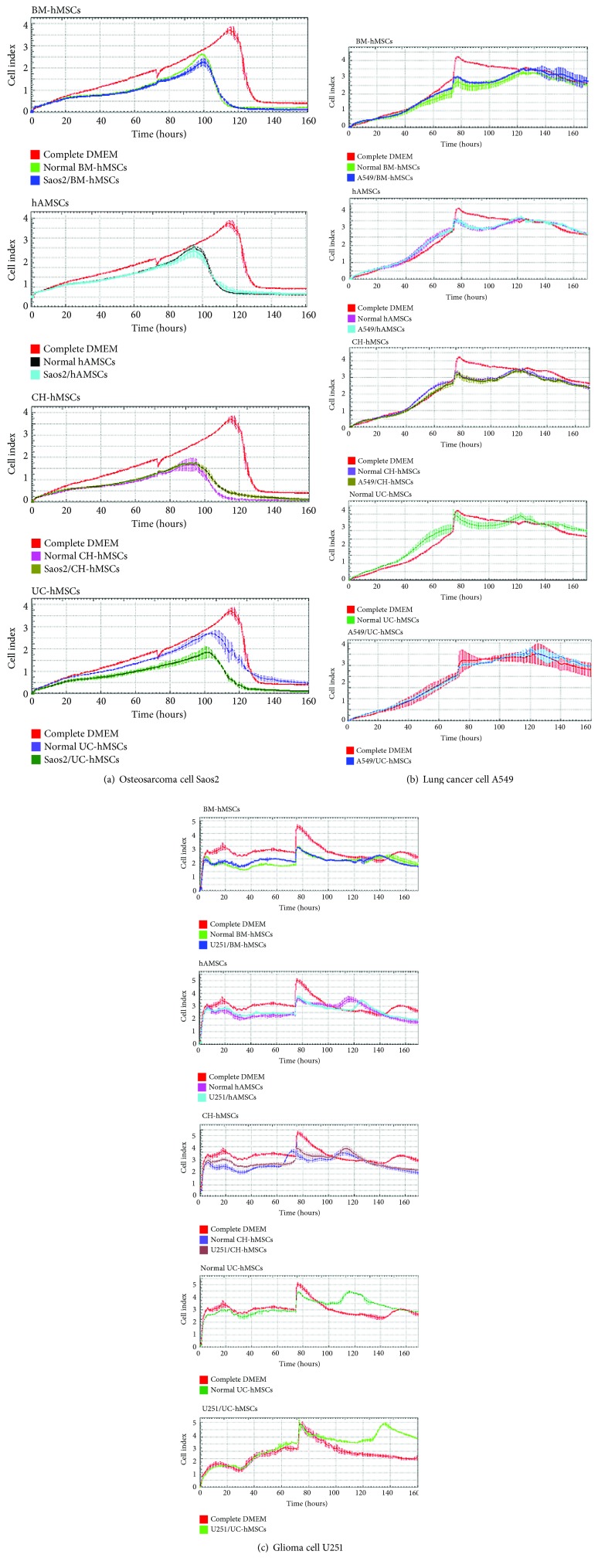
Effects of hMSC-derived bioactive molecules on the proliferation of Saos2, A549, and U251 cells. Graphs show the growth kinetics of osteosarcoma cell Saos2 (a), lung adenocarcinoma cell A549 (b), and glioma cell U251 (c), which were cultured in conditioned medium derived from various normal and cancer-associated hMSC-conditioned media for 7 days. The growth kinetics of cancer cells were determined by the xCELLigence real-time cell analyzer and were reported as the cell index against culture time. Saos2, A549 and U251 cells cultured in DMEM supplemented with 10% (*v*/*v*) FBS serve as controls. Data are presented as the mean ± SEM of three independent experiments.

Although bone marrow- and gestational tissue-derived hMSCs decreased the proliferation of osteosarcoma cell Saos2, their inhibitory effects on Saos2 cells appeared to be lesser in degree and later in onset compared with their effects on C3A and HT29 cells (Figure 3(a)). In contrast to C3A, HT29, and Saos2 cells, all hMSCs failed to inhibit the proliferation of lung adenocarcinoma cell A549 and glioma cell U251 (Figures 3(b) and 3(c)).

To determine whether cancer-derived bioactive molecules can change the cancer suppressive ability of hMSCs, bone marrow- and gestational tissue-derived hMSCs were co-cultured with various cancer cells using transwell culture system for 7 days before the conditioned media derived from those hMSCs (called “cancer-associated hMSC-conditioned media”) were collected and used for cancer cell proliferation assay. The results showed that the cancer-associated hMSC-conditioned media exhibited the same levels of cancer suppressive capacity compared with their normal hMSC counterparts (Figures 2 and 3).

Those results demonstrate that the bioactive molecules secreted from bone marrow- and gestational tissue-derived hMSCs exerted different effects on distinct human cancer cells and cancer-derived bioactive molecules did not affect the cancer suppressive capacity of hMSCs. According to this, only C3A and HT29, whose proliferation was clearly suppressed by hMSC-derived bioactive molecules, were chosen for the further study.

### 3.3. Effect of Fractionated hMSC Secretome on C3A and HT29 Cell Proliferation

To further characterize the hMSC-derived factors that inhibited C3A and HT29 cell proliferation, the conditioned media derived from normal BM-hMSCs, hAMSCs, CH-hMSCs, and UC-hMSCs were fractionated to 5 distinct fractions according to the molecular weight of their protein compositions. The effects of each hMSC-conditioned media fraction on C3A and HT29 cell proliferation were then determined and compared with their unfractionated hMSC-conditioned media.

**Figure 4:**
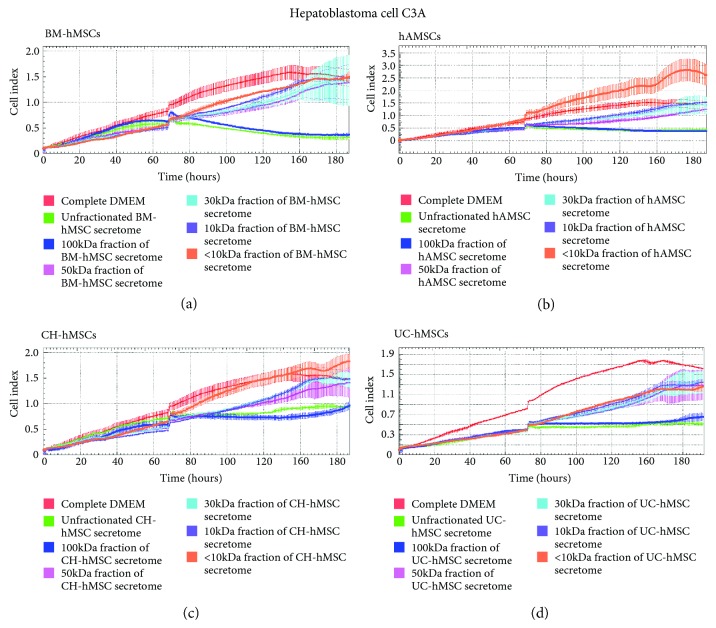
Effects of various fractions of hMSC secretomes on the proliferation of C3A cells. Graphs show the growth kinetics of hepatoblastoma cell C3A which were cultured in the 100 kDa, 50 kDa, 30 kDa, 10 kDa, and less than 10 kDa (<10 kDa) fraction of the conditioned media derived from BM-hMSCs (a), hAMSCs (b), CH-hMSCs (c), and UC-hMSCs (d) for 7 days. The growth kinetics of C3A cells were determined by the xCELLigence real-time cell analyzer and were reported as the cell index against culture time. C3A cells cultured in DMEM supplemented with 10% (*v*/*v*) FBS serve as negative controls while C3A cells cultured in unfractionated hMSC-conditioned media serve as positive controls. Data are presented as the mean ± SEM of three independent experiments.

The results showed that only the 100 kDa fraction of BM-hMSC-, hAMSC-, CH-hMSC-, and UC-hMSC-conditioned media reduced the proliferation of C3A cells to the same levels as those cultured in the unfractionated hMSC-conditioned media (Figure 4(a)–(d)). Although the 50 kDa, 30 kDa, and 10 kDa fraction of BM-hMSC-, hAMSC-, CH-hMSC-, and UC-hMSC-conditioned media also reduced the proliferation of C3A cells in comparison to controls; their inhibitory effects were much lesser than the 100 kDa fraction and unfractionated hMSC-condition media (Figure 4(a)–(d)).

**Figure 5:**
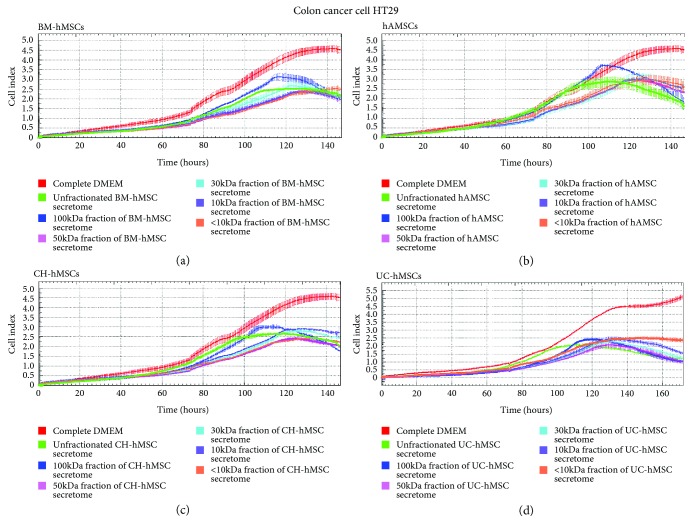
Effects of various fractions of hMSC secretomes on the proliferation of HT29 cells. Graphs show the growth kinetics of colon adenocarcinoma cell HT29 which were cultured in the 100 kDa, 50 kDa, 30 kDa, 10 kDa, and less than 10 kDa (<10 kDa) fraction of the conditioned media derived from BM-hMSCs (a), hAMSCs (b), CH-hMSCs (c), and UC-hMSCs (d) for 7 days. The growth kinetics of HT29 cells were determined by the xCELLigence real-time cell analyzer and were reported as the cell index against culture time. HT29 cells cultured in DMEM/F12 supplemented with 10% (*v*/*v*) FBS serve as negative controls while HT29 cells cultured in unfractionated hMSC-conditioned media serve as positive controls. Data are presented as the mean ± SEM of three independent experiments.

While the inhibitory effects of the 100 kDa fraction of BM-hMSC-, hAMSC-, CH-hMSC-, and UC-hMSC-conditioned media on C3A cell proliferation were greatest (Figure 4(a)–(d)), their ability to suppress HT29 cell proliferation were the lowest in comparison to other fractions (Figure 5(a)–(d)). It appeared that the ability to suppress HT29 cell proliferation of 50 kDa, 30 kDa, 10 kDa, and <10 kDa fraction of BM-hMSC-, hAMSC-, CH-hMSC-, and UC-hMSC-conditioned media was greater than the 100 kDa fraction (Figure 5(a)–(d)). The results clearly indicate that the hMSC-derived factors which inhibit C3A cell proliferation were distinct from those that inhibit HT29 cell proliferation.

### 3.4. Effect of hMSC-Derived Bioactive Molecules on C3A Cell Migration

**Figure 6:**
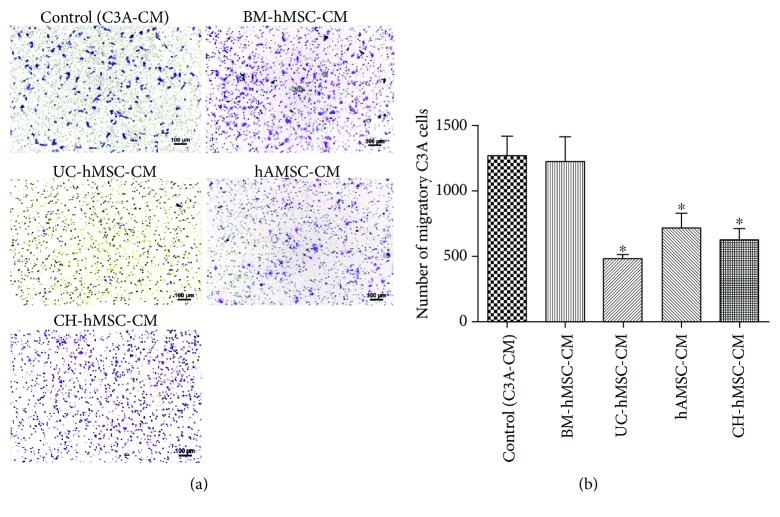
The effect of hMSC-derived bioactive molecules on C3A cell migration. (a) C3A cells which migrated to the other side of transwell membrane after being induced by various hMSC-conditioned media as determined by hematoxylin staining. Scale bar: 100 *μ*m. C3A cells cultured with C3A-conditioned medium serve as controls. (b) Graph shows the number of migratory C3A cells after induction with various hMSC-conditioned media. Data are presented as the mean ± SEM of three independent experiments. One-way ANOVA was used to assess the significance of differences between observed data. ^∗^ *P* < 0.05 vs. controls.

To study the effect of hMSC-derived bioactive molecules on C3A cell migration, the conditioned media derived from various hMSC sources were used to induce C3A cell migration through 8 *μ*m transwells. Interestingly, the bioactive molecules secreted from UC-hMSCs, hAMSCs, and CH-hMSCs induced a significantly lower number of migratory C3A cells (471 ± 31 cells, 709 ± 123 cells, and 632 ± 83 cells, respectively) in comparison to controls (1268 ± 143 cells, *P* < 0.05) (Figures 6(a) and 6(b)). In contrast, the number of migratory C3A cells that were induced by bioactive molecules released from BM-hMSCs (1227 ± 174 cells) was not different from controls (1268 ± 143 cells) (Figures 6(a) and 6(b)). The results suggest that while the bioactive molecules secreted from UC-hMSCs, hAMSCs, and CH-hMSCs suppressed C3A cell migration, the BM-hMSC-derived bioactive molecules did not have this effect.

### 3.5. Migratory Ability of hMSCs toward C3A and HT29 Cells

To study the migratory ability of bone marrow- and gestational tissue-derived hMSCs toward C3A and HT29 cells, BM-hMSCs, hAMSCs, CH-hMSCs, and UC-hMSCs were co-cultured with C3A or HT29 cells using the transwell culture system. The numbers of hMSCs that migrated toward C3A or HT29 were then determined after 6 hours of co-culture.

**Figure 7:**
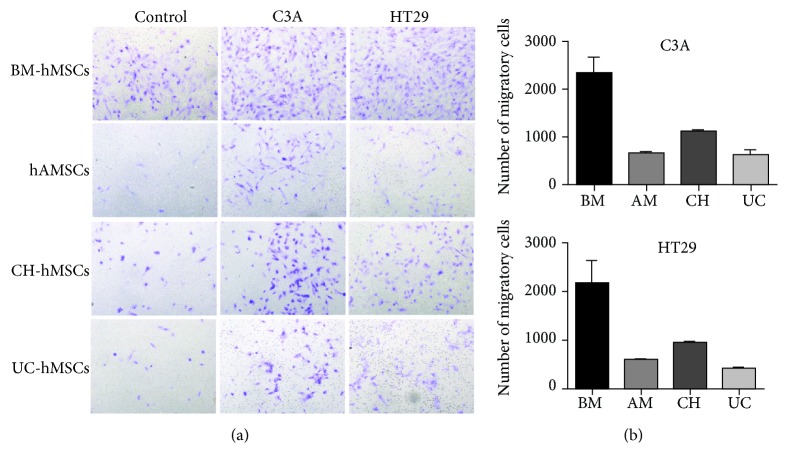
Migratory ability of hMSCs toward C3A and HT29 cells. (a) Hematoxylin-stained hMSCs which migrated to the other side of transwell membrane in response to bioactive molecules released from C3A and HT29 cells. hMSCs cultured in transwells containing DMEM supplemented with 2% (*v*/*v*) FBS serve as controls. (b) The number of hMSCs which migrated in response to bioactive molecules released from C3A and HT29 cells. Data are presented as the mean ± SEM of three independent experiments.

The results showed that distinct sources of hMSCs exhibited different migratory abilities toward C3A and HT29 cells. BM-hMSCs have greater migratory ability toward C3A cells (2311 ± 348 cells) than CH-hMSCs (1124 ± 28 cells), hAMSCs (660 ± 21 cells), and UC-hMSCs (625 ± 81 cells) (Figures 7(a) and 7(b)). Similar to C3A, BM-hMSCs also exhibited greater migratory ability toward HT29 cells (2161 ± 434 cells) than CH-hMSCs (946.5 ± 41 cells), hAMSCs (592 ± 34 cells), and UC-hMSCs (415 ± 4 cells) (Figures 7(a) and 7(b)).

### 3.6. Identification of Proteins Presented in the 100 kDa Fraction of hAMSC-Conditioned Medium

To identify hMSC-derived factors that were able to suppress C3A cell proliferation, we chose the 100 kDa fraction of hAMSC-conditioned medium, which was shown to suppress the proliferation of C3A cells, for mass spectrometry analysis.

We chose hAMSC-conditioned medium for further analysis based on two reasons. Firstly, despite BM-hMSCs exhibiting similar levels of cancer suppressive effect, their proliferative capacity in culture is lesser than hAMSCs and could not be expanded for more than 10 passages. Because the preparation of hMSC-derived bioactive factors for future clinical applications required a large number of cultured hMSCs, we believe that hAMSCs which can be isolated from placental tissues in large quantity and can be expanded for a longer period of time than BM-hMSCs will be more suitable for this purpose. Secondly, our previous study demonstrates that hAMSCs release other therapeutically useful factors that induce migration of endothelial progenitor cells which play important roles in tissue neovascularization while other gestational tissue-derived hMSCs, such as UC-hMSCs, did not [23, 24].

**Figure 8:**
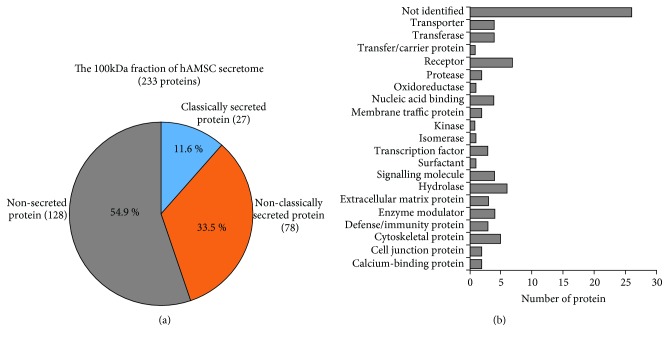
Characterization of the 100 kDa fraction of hAMSC secretome. (a) Pie chart illustrates 233 proteins presented in the 100 kDa fraction of hAMSC secretome as being categorized into non-secreted protein, classically secreted protein, and non-classically secreted protein. (b) Graph demonstrates secretory proteins presented in the 100 kDa fraction of hAMSC secretome as being categorized into groups according to their functions.

The results showed that the 100 kDa fraction of hAMSC-conditioned medium consisted of 233 proteins, of which 128 were non-secreted proteins, 78 were non-classically secreted proteins, and 27 were classically secreted proteins (Figure 8(a)). To identify hMSC-derived bioactive molecules that suppress C3A cell proliferation, 128 non-secreted proteins that might be released from dead hAMSCs during the preparation of the conditioned medium were excluded while the rest of the proteins were categorized based on their functions (Figure 8(b)). Lists of bioactive molecules identified in the 100 kDa fraction of hAMSC secretome are provided in supplementary table 1.

## 4. Discussion

hMSCs have been considered the promising sources for cell therapy. The therapeutic potentials of hMSCs depend largely on their ability to secrete a broad range of bioactive molecules that affect various processes, such as immune response, cell proliferation, and neovascularization [25]. This is in agreement with our pervious study which demonstrates that the hAMSCs and BM-hMSCs release proangiogenic factors, such as PDGF-*β*, IGF-1, and SDF-1, which increase migration, invasion, and vessel-forming capacity of endothelial progenitor cells (EPCs) [23]. EPCs have been shown to play important roles in tumor neovascularization [26–28], and hence, our previous study suggests that hMSCs might indirectly affect tumor growth by regulating EPC functions.

Apart from affecting EPC functions, several previous studies show that hMSCs enhanced engraftment of several cancers, including breast cancer, ovarian cancer, melanoma, glioma, and colon cancer cells [9, 10, 12, 13, 16, 17, 29–32]. They have also been shown to be the source of cancer-associated fibroblasts (CAFs) that enhance tumor growth and metastasis by secreting various bioactive molecules that induce tumor neovascularization and inhibit immune reaction against cancer cells [8–17]. Contrary to those evidences, however, several other studies showed that hMSC inhibited growth and metastasis of several cancers, including colon cancer, hepatocellular carcinoma, and melanoma in animal models [19, 20, 33]. Those conflicting results possibly arise from the variability in both hMSC sources and cancer cell types used in those studies.

Indeed, the present study showed that the effects of hMSC-derived bioactive molecules on cancer cell proliferation depend on both hMSC sources and cancer cell types. Some particular cancer cells, such as hepatoblastoma cell C3A and colon adenocarcinoma cell HT29, were very sensitive to the suppressive effects of hMSCs while the proliferation of lung adenocarcinoma cell A549 was not affected by hMSC-derived bioactive molecules. The insensitivity of A549 to hMSC-derived factors is in agreement with the previous study which showed that Wharton's jelly-derived hMSC (WJ-hMSC) secretome and human amniotic membrane protein extract (hAMPE) did not affect the proliferation of A549 cells [34]. Also in agreement with the previous study showing that amniotic membrane-derived hMSCs (AM-hMSCs) suppressed the proliferation of Saos2 cells [35], we observed the inhibitory effects of both bone marrow and gestational tissue-derived hMSCs on Saos2 cell proliferation. However, the inhibitory effects of hMSCs on Saos2 cells appeared to be lesser in degree and later in onset compared with their effects on C3A and HT29 cells.

When compared among distinct hMSC sources, hAMSCs and CH-hMSCs inhibited cancer cell proliferation at the level similar to that of BM-hMSCs, which is the standard source of hMSCs for clinical applications. Interestingly, while BM-hMSCs have greater migratory ability toward cancer cells than gestational tissue-derived hMSCs, their ability to suppress hepatoblastoma cell migration was lower than those of gestational tissue-derived hMSCs.

Although several previous studies showed that cancer-derived bioactive molecules could induce hMSCs to release bioactive molecules that promote cancer cell proliferation [36–38], we found that cancer-derived bioactive molecules, regardless of both hMSC sources and cancer cell types, did not affect the cancer suppressive capacity of hMSCs.

The hMSC-derived factors that inhibit the proliferation of hepatoblastoma cell C3A were distinct from those that inhibited the proliferation of colon adenocarcinoma cell HT29. The hMSC-derived factors that suppress the proliferation of C3A cells, regardless of hMSC sources, were clearly enriched in the 100 kDa fraction of hMSC-conditioned media while hMSC-derived factors that suppress HT29 proliferation, also regardless of the hMSC sources, were enriched in the 50 kDa, 30 kDa, and 10 kDa fractions of hMSC-conditioned media. The results suggest that all hMSCs might suppress the proliferation of C3A and HT29 cells by secreting the same combination of bioactive molecules (in which the bioactive molecules that suppress C3A cell proliferation were mostly large proteins whose molecular weights exceed 100 kDa while the bioactive molecules that suppress HT29 proliferation were consisted of several proteins that have distinct molecular weights).

To identify hMSC-derived factors that were able to suppress C3A cell proliferation, we chose the 100 kDa fraction of hAMSC-conditioned medium, which was shown to be enriched for factors that suppress the proliferation of C3A cells, for mass spectrometry analysis. The results revealed that the 100 kDa fraction of hAMSC secretome consisted of at least 105 secreted bioactive molecules that belong to various groups of proteins, such as extracellular matrix, proteinase, and cytokines together with novel proteins whose functions have not been identified. Among the identified bioactive molecules are proteins that have previously been shown to suppress the proliferation of various cancer cells, including HEAT repeat-containing protein 1 (HEATR1) [39]; leprecan-like protein (LEPREL) [40, 41]; NK1 transcription factor-related protein 2 (NK1.2) [42]; small inducible cytokine subfamily E, member 1 (SCYE1 or AIMP1) [43, 44]; sushi domain-containing protein 2 (SUSD2) [45]; arylsulfatase B (ARSB) [46]; and type IV collagen [47] (Supplementary table 1).

Compared with ELISA array and western blot analysis that has been used to identify limited a number of hMSC-derived factors in the previous studies [48–50], the mass spectrometry analysis used in our study could discover complete protein composition of hAMSC secretome in an unbiased manner. As a result, we have identified several factors which have not been described in the previous literatures. However, due to the large number of bioactive molecules presented in the 100 kDa fraction of hAMSC-conditioned medium, the further fractionation of hMSC-conditioned media into smaller subfractions is required for the identification of the exact hMSC-derived factors that suppress cancer cell proliferation.

## 5. Conclusion

We herein report that the effects of hMSCs on cancer cell proliferation depend on both hMSC sources and cancer cell types. We also demonstrated that cancer-derived bioactive molecules did not affect the cancer suppressive capacity of hMSCs. Moreover, hMSCs might use a distinct combination of bioactive molecules to suppress the proliferation of human hepatoblastoma and colorectal cancer cells. We believe that the *in vitro* system developed in this study could be used to discover other therapeutically useful molecules released from hMSCs for the future *in vivo* study.

## Data Availability

The datasets used and/or analyzed during the current study are available from the corresponding author on reasonable request.

## Abbreviations

BM-hMSCs: Bone marrow-derived human mesenchymal stem cells
CH-hMSCs: Chorion-derived human mesenchymal stem cells
hAMSCs: Placenta-derived human mesenchymal stem cells
UC-hMSCs: Umbilical cord-derived human mesenchymal stem cell
Complete DMEM: DMEM supplemented with 10% (*v*/*v*) fetal bovine serum
Normal hMSCs: Conditioned media derived from hMSCs
C3A/hMSCs: Conditioned media derived from hMSCs co-cultured with C3A cells for 7 days
HT29/hMSCs: Conditioned media derived from hMSCs co-cultured with HT29 cells for 7 days
Saos2/hMSCs: Conditioned media derived from hMSCs co-cultured with Saos2 cells for 7 days
A549/hMSCs: Conditioned media derived from hMSCs co-cultured with A549 cells for 7 days
U251/hMSCs: Conditioned media derived from hMSCs co-cultured with U251 cells for 7 days.

## Additional Points

*Novelty and Impact Statements.* We herein demonstrate that the effect of hMSCs on cancer cell proliferation and migration depends on both hMSC sources and cancer cell types. We also showed that cancer-derived bioactive molecules did not affect the cancer suppressive capacity of hMSCs and each hMSC use distinct combination of bioactive molecules to suppress the proliferation of human hepatoblastoma and colorectal cancer cells. The procedure developed in this study could be used to discover other therapeutically useful molecules produced by hMSCs for future research and clinical uses.

## Supplementary Materials

Supplementary Materials List of secreted proteins in the 100 kDa fraction of hAMSC secretome as identified by mass spectrometry analysis in Figures 7(a) and 7(b).
