# Supplementary material for: Gestational Tissue-Derived Human Mesenchymal Stem Cells Use Distinct Combinations of Bioactive Molecules to Suppress the Proliferation of Human Hepatoblastoma and Colorectal Cancer Cells
Source: Stem Cells Int. 2019 Jul 4;2019:9748795. doi: 10.1155/2019/9748795 (PMC6637692; doi:10.1155/2019/9748795)
Supplement: Supplementary Materials — List of secreted proteins in the 100 kDa fraction of hAMSC secretome as identified by mass spectrometry analysis in Figures 7(a) and 7(b). [file 9748795.f1.docx]

**Supplementary table 1**

Lists of secreted bioactive molecules identified in the 100 kDA fraction of hAMSC secretome

| Protein name | Accession  number | SignalP | SecretomeP | PANTHER classification |
| --- | --- | --- | --- | --- |
| ADAM metallopeptidase with thrombospondin type 1 motif | gi\|119631213 | Yes |  | Protease |
| A type IV collagen | gi\|15991848 | Yes |  | Extracellular matrix |
| Antigen MLAA-21 | gi\|37574654 |  | Yes | Cell junction protein  G-protein modulator |
| Heparan-sulfate 6-O-sulfotransferase 2 isoform L | gi\|116295256 |  | Yes | Transferase |
| Motile sperm domain-containing protein 1 | gi\|9506543 |  | Yes | - |
| Astrotactin-1 | gi\|46488923 | Yes |  | Cell junction protein |
| Phospholipid-transporting ATPase IB | gi\|117168245 |  | Yes | Hydrolase  Transporter |
| Polyamine-modulated factor 1 isoform 3 | gi\|313851053 |  | Yes | - |
| RCSD1 protein | gi\|48735391 |  | Yes | Cytoskeletal protein |
| SLIT-ROBO Rho GTPase activating protein 2 | gi\|55662261 |  | Yes | - |
| MSTP018 (Integrin alpha-11) | gi\|17432223 |  | Yes | Cell adhesion/Receptor |
| Mutant CD8 alpha antigen | gi\|14861040 | Yes |  | Defense/immunity protein |
| Nebulette | gi\|3660517 |  | Yes | - |
| Apical protein 2, isoform CRA_a | gi\|119582714 |  | Yes | - |
| Arylsulfatase B | gi\|71852584 | Yes |  | Hydrolase |
| B-cell CLL/lymphoma 7 protein family member B isoform 2 | gi\|308522773 |  | Yes | - |
| C1q and tumor necrosis factor related protein 3 variant | gi\|62088102 |  | Yes | - |
| C3 and PZP-like alpha-2-macroglobulin domain containing 8 | gi\|23379807 | Yes |  | Signaling molecule |
| Chain A, Crystal Structure Of The Mammalian Copii-coat Protein Sec23/24 Bound To The Transport Signal Sequence Of Membrin | gi\|209870512 |  | Yes | G-protein modulator |
| Chain A, Crystallographic And Kinetic Studies Of Human Mitochondrial Acetoacetyl-coa Thiolase (t2): The Importance Of Potassium And Chloride For Its Structure And Function | gi\|145579614 |  | Yes | Acetyltransferase |
| Chain A, Hr1b Domain From Prk1 | gi\|159163170 |  | Yes | Kinase  Carrier protein |
| General transcription factor IIF, polypeptide 1, 74kDa, isoform CRA_b | gi\|119589505 |  | Yes | Transcription factor binding |
| HEATR1 protein | gi\|15080480 |  | Yes | - |
| Histidine triad nucleotide binding protein 2, isoform CRA_c | gi\|119578737 |  | Yes | - |
| Chain A, Solution Structure Of The Death Domain Of Ankyrin-1 | gi\|171848938 |  | Yes | Cytoskeletal protein |
| Chain A, The Three-Dimensional Structure Of The C-Terminal Dna Binding Domain Of Human Ku70 | gi\|15988053 |  | Yes | Nucleic acid binding |
| Chain A, X-Ray Structure Of Human Phosphomannomutase 2 (Pmm2) | gi\|75766280 |  | Yes | Isomerase |
| Chain B, Crystal Structure Of The Human Taf4-Taf12 (Tafii135-Tafii20) Complex | gi\|24158663 |  | Yes | - |
| Chaperonin containing TCP1, subunit 4 (delta), isoform CRA_a | gi\|119620390 |  | Yes | Nucleotide binding |
| Chondroitin sulfate synthase 1 precursor | gi\|31542309 | Yes |  | Glycosyltransferase |
| EP3-V | gi\|2114191 |  | Yes | G-protein coupled receptor |
| ERI1 exoribonuclease 3 isoform 1 | gi\|74136559 |  | Yes | Hydrolase |
| Ig kappa chain V-III region (Sca) - human (fragment) | gi\|106611 |  | Yes | - |
| DEC-205/DCL-1 fusion protein variant V34-2 | gi\|32307817 | Yes |  | Receptor |
| Delta-like protein 1 precursor | gi\|110735443 | Yes |  | Cell signaling |
| Down syndrome cell adhesion molecule isoform CHD2-42 precursor variant | gi\|62087852 | Yes |  | - |
| Immunoglobulin kappa light chain variable region | gi\|116795057 |  | Yes | - |
| Immunoglobulin lambda chain variable region | gi\|587390 |  | Yes | - |
| Immunoglobulin light chain variable region | gi\|13549148 |  | Yes | - |
| Immunoglobulin variable region | gi\|323431845 |  | Yes | - |
| KIAA0391 | gi\|27882031 |  | Yes | - |
| KIAA0976 protein | gi\|40789006 |  | Yes | Receptor/ ECM linker protein |
| Coiled-coil domain containing 78, isoform CRA_f | gi\|119606147 |  | Yes | - |
| Cystine/glutamate transporter | gi\|7657683 |  | Yes | Amino acid transporter |
| Immunoglobulin gamma 2 heavy chain variable region | gi\|304562513 |  | Yes | - |
| LDLR-FUT fusion protein | gi\|6739500 | Yes |  | - |
| Leprecan-like protein | gi\|119609139 |  | Yes | Extracellular matrix organization |
| NK1 transcription factor-related protein 2 | gi\|226437602 |  | Yes | Nucleic acid binding  Transcription factor |
| MHC class II antigen | gi\|84796223 | Yes |  | Immunity |
| CD95 ligand (Tumor necrosis factor) | gi\|61658441 |  | Yes | - |
| Immunoglobulin variable region | gi\|323431845 |  | Yes | - |
| KIAA0391 | gi\|27882031 |  | Yes | - |
| KIAA0976 protein | gi\|40789006 |  | Yes | Receptor/ ECM linker protein |
| KIF6 protein | gi\|109658866 |  | Yes | Cytoskeletal protein |
| Laminin, beta 3, isoform CRA_a | gi\|119613854 | Yes |  | ECM linker protein |
| Solute carrier family 6, member 15, isoform CRA_a | gi\|119617794 |  | Yes | Ion transport |
| Sushi domain-containing protein 2 | gi\|10092665 | Yes |  | Immnunity/cytokine |
| Synaptotagmin-10 | gi\|39752671 |  | Yes | Membrane trafficking regulatory protein |
| RecName: Full=Zinc finger and BTB domain-containing protein 44 | gi\|74760158 |  | Yes | Transcription cofactors |
| SEMA6C protein | gi\|92058719 | Yes |  | Signaling molecule |
| Semaphorin receptor | gi\|6010211 | Yes |  | Receptor |
| PILR alpha-associated neural protein isoform a precursor | gi\|24308547 | Yes |  | Immunity molecule |
| Plasminogen | gi\|38051823 | Yes |  | Peptide hormone/receptor/serine proteinase |
| Pyruvate dehydrogenase phosphatase regulatory subunit | gi\|152013038 |  | Yes | Oxidoreductase |
| SETMAR protein, partial | gi\|33869529 |  | Yes | DNA binding |
| Seven transmembrane helix receptor | gi\|21928448 |  | Yes | - |
| Small inducible cytokine subfamily E, member 1 | gi\|119626608 |  | Yes | Cytokine activity |
| Syntaxin 16 | gi\|2961087 |  | Yes | Membrane trafficking regulatory protein |
| TAF15 | gi\|1373378 |  | Yes | - |
| TBC1 domain family, member 7 variant | gi\|62898451 |  | Yes | - |
| WD repeat domain 35 | gi\|22477171 |  | Yes | - |
| Zinc finger protein 449 | gi\|194239638 |  | Yes | Nucleic acid binding |
| Tumor necrosis factor receptor superfamily member 8 isoform 1 precursor | gi\|597709795 | Yes |  | Receptor |
| Ubiquitously transcribed tetratricopeptide repeat protein Y-linked transcript variant 283 | gi\|151946833 |  | Yes | Transcription factor |
| Ubiquitously transcribed tetratricopeptide repeat protein Y-linked transcript variant 4 | gi\|148733168 |  | Yes | Transcription factor |
| Phosphodiesterase isozyme 7 | gi\|30421104 |  | Yes | Signal transduction |
| Serum amyloid A protein beta des-Arg(pI5.6), SAA1 beta des-Arg pI5.6 | gi\|247142 |  | Yes | - |
| Voltage-gated calcium channel alpha(2)delta-4 subunit | gi\|22770594 |  | Yes | Calcium-binding protein |
